# Association between thyroidea function and opioid agonist therapy: a prospective cohort study from Norway

**DOI:** 10.1210/jendso/bvag079

**Published:** 2026-04-07

**Authors:** Anne Taraldsen Heldal, Jorn Henrik Vold, Kristin Viste, Fatemeh Chalabianloo, Gunnar Mellgren, Kjell Arne Johansson, Lars Thore Fadnes

**Affiliations:** Bergen Addiction Research and Norwegian Research Center for Agonist Treatment of Substance Use Disorder, Addiction and Substance Use Clinic, Haukeland University Hospital, 5009 Bergen, Norway; Department of Community Medicine and Global Health, University of Oslo, 0450 Oslo, Norway; Bergen Addiction Research and Norwegian Research Center for Agonist Treatment of Substance Use Disorder, Addiction and Substance Use Clinic, Haukeland University Hospital, 5009 Bergen, Norway; Department of Global Public Health and Primary Care, University of Bergen, 5009 Bergen, Norway; Division of Psychiatry, Haukeland University Hospital, 5009 Bergen, Norway; Hormone Laboratory, Department of Medical Biochemistry and Pharmacology, Haukeland University Hospital, 5009 Bergen, Norway; Department of Clinical Science, University of Bergen, 5009 Bergen, Norway; Bergen Addiction Research and Norwegian Research Center for Agonist Treatment of Substance Use Disorder, Addiction and Substance Use Clinic, Haukeland University Hospital, 5009 Bergen, Norway; Department of Clinical Medicine, University of Bergen, 5009 Bergen, Norway; Hormone Laboratory, Department of Medical Biochemistry and Pharmacology, Haukeland University Hospital, 5009 Bergen, Norway; Department of Clinical Science, University of Bergen, 5009 Bergen, Norway; Bergen Addiction Research and Norwegian Research Center for Agonist Treatment of Substance Use Disorder, Addiction and Substance Use Clinic, Haukeland University Hospital, 5009 Bergen, Norway; Department of Global Public Health and Primary Care, University of Bergen, 5009 Bergen, Norway; Bergen Addiction Research and Norwegian Research Center for Agonist Treatment of Substance Use Disorder, Addiction and Substance Use Clinic, Haukeland University Hospital, 5009 Bergen, Norway; Department of Global Public Health and Primary Care, University of Bergen, 5009 Bergen, Norway

**Keywords:** opioid substitution treatment, thyroid function, substance-related disorders, injecting substance use

## Abstract

**Background:**

Opioid agonist therapy (OAT) is an essential treatment for opioid dependency. However, OAT may lead to endocrinopathy, which may impair the tolerability of treatment. Although the risk of disturbed regulation of the adrenal and gonadal axes has been extensively studied, less is known about how long-term opioid use and corresponding risk factors influence the thyroid axis. The objective of this study was to investigate associations between biomarkers of thyroid function and OAT medication as well as sociodemographic and clinical factors and concurrent use of illicit substances among patients receiving OAT.

**Methods:**

We used prospective data from 320 people receiving OAT in Bergen, Norway, from 2016 through 2023. All had 2 health assessments, including serum measurements of TSH. Descriptive statistics and a linear mixed model with coefficient and 95% CIs were performed to investigate the association between serum TSH (measured in mIU/L) and OAT medication as well as age, sex, substance use patterns, injecting use, housing status, and educational attainment at first assessment and over time.

**Results:**

Median serum TSH at first assessment in the study population was 1.8 mIU/L (interquartile range: 1.2). No association between OAT medications, sociodemographic, or clinical factors and serum TSH was found at first assessment and over time.

**Conclusion:**

The median serum TSH was within the recommended range among people receiving OAT. No association between OAT treatment and thyroid insufficiency was found. This indicates that screening for thyroid function beyond symptomatic assessment is probably not needed among patients receiving OAT.

Opioid dependence is a complex illness characterized by several criteria, including a strong desire to use opioids, severe symptoms when discontinued, and increased opioid tolerance. Opioid agonist therapy (OAT) with methadone, buprenorphine, or buprenorphine-naloxone is a recommended and well-established treatment for opioid use disorder [1]. Evidence of its efficacy has been demonstrated with substantially reduced mortality, somatic comorbidity, criminal convictions, and risk of fatal overdoses [2, 3]. Hence, OAT is a cost-effective treatment that generates less economic burden on society, including reduced costs for health care, social welfare, and legal services [3-7]. However, there are some potentially adverse effects of long-term use of opioids, including how opioids impact the endocrine systems [8]. Of these, opioid-induced secondary (hypothalamic/pituitary) hypogonadism has been most extensively studied, although the prevalence remains unclear [9-12]. The effects of opioids on the hypothalamic pituitary adrenal axis are also well-known [8, 13]; however, little is known about how opioids impact the hypothalamic-pituitary-adrenal axis among patients receiving OAT, who often have complex somatic and psychiatric comorbidities and use illicit substances.

In most cases, the recommended initial test for thyroid function is serum TSH. The thyroid gland hormones, T4 and T3, have several important roles in regulating metabolism, including, among others, involvement in normal cell function and growth, protein synthesis, lipid and carbohydrate metabolism, calcium homeostasis, thermogenesis, brain and muscle activity, and myocardial contractility [14]. The pituitary hormone TSH stimulates the synthesis of T3 and T4 through a negative feedback loop where the production of TSH is attenuated by increased T3 and T4, both through direct negative feedback and indirectly on the hypothalamic thyrotropin releasing hormone.

T4 more accurately reflects the functioning of thyroid gland because it is bound to proteins that prevent it from entering the thyroid tissue [15]. Measuring serum T4 is useful when TSH is outside the recommended reference interval, when there is clinical suspicion of pituitary insufficiency, or when monitoring patients receiving initial treatment for thyroid disorders [16].

In patients receiving OAT, there are potentially increased risks of endocrinopathy also on thyroid function because of the specific clinical characteristics of this patient group. The opioid doses used in OAT are significantly higher than opioid doses used in other patient groups treated with long-term opioids [17, 18]. Use of illicit drugs, including injection drug use, is higher compared to the general population [19], and there is a higher burden of somatic comorbidities [20] and malnutrition [21, 22]. Few studies have investigated this potential opioid induced endocrinopathy in patients receiving OAT. Thus, the objective for this study was to assess a possible association between long-term use of opioids in patients receiving OAT, including clinical and sociodemographic characteristics and thyroid function.

## Materials and methods

### Study characteristics: design, population, data collection, and study sample

This is a prospective longitudinal cohort study presenting data drawn from the multicenter INTRO-HCV and ATLAS4LAR studies [23-26]. The study sample included patients receiving OAT in Bergen, Norway, from 2016 to 2023. All patients were older than 18 years, met the criteria for opioid dependence syndrome according to the International Classification of Diseases, version 10, and received methadone, buprenorphine-based medication, or morphine daily according to the OAT program. The patients underwent their annual assessment at a time when they were not abstinent from substances, yet did not exhibit overt signs of intoxication and were able to engage cooperatively in both the interview and the clinical examination.

All included patients had an annual health assessment by trained research nurses performing questionnaire-based interviews that focused on several somatic, mental health, and psychosocial aspects, including substance use patterns. Data were collected using the software CheckWare (CheckWare, Trondheim, Norway). Clinical data were obtained from the electronic medical record. Patients who carried out 2 health assessments, including measurements of serum TSH, were included [27].

**Figure 1:**
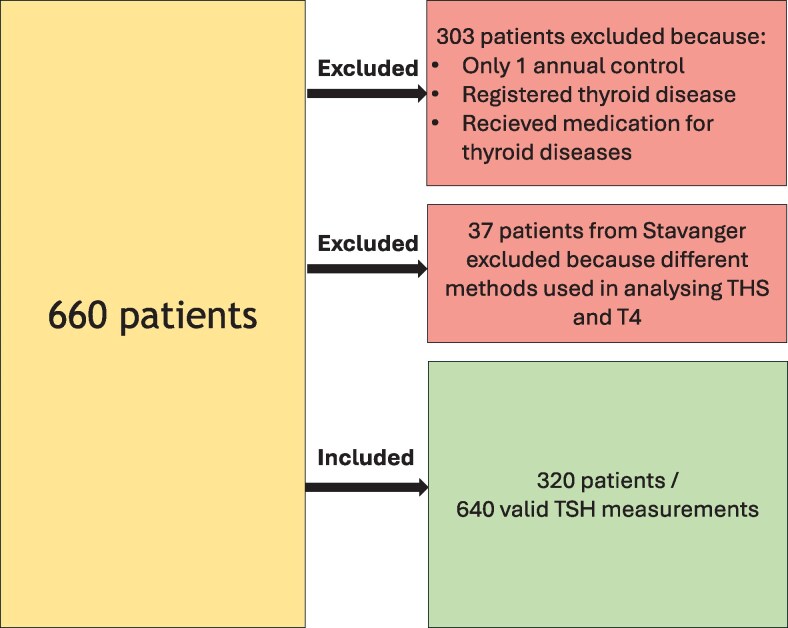
Flow chart demonstrating inclusion and exclusion criteria of patients.

Information on endocrine, nutritional, and metabolic diseases registered in the specialized health care program were collected, derived from International Classification of Diseases, version 10, chapter 4 (E00-E90) diagnose codes. Patients with registered thyroid disease (E03 or E05) were excluded. In addition, all Norwegian residents had a summary care record providing information of all medications dispensed by pharmacies, from both electronic and paper prescriptions for the past 3 years [28]. To identify patients who had been diagnosed and treated in primary care beyond specialized health care, patients who received medication for thyroid diseases were excluded by information retrieved from the summary care record. Hence, all patients with a registered thyroid disease and patients receiving medication for thyroid diseases were excluded from the entire study period. Data were collected between January 2017 and August 2023. A total of 320 patients with a total of 640 valid serum TSH and T4 measurements were included (Fig. 1). The mean time between the first and second health assessment was 1.72 years (SD: 0.83). The first serum measurement of TSH was defined as the first assessment, whereas the subsequent measurement was defined as the second assessment.

### Biochemical analyses

Venous blood samples were collected and analyzed for serum TSH, T4, C-reactive protein (CRP), and albumin at the Department of Medical Biochemistry and Pharmacology Haukeland University Hospital in Bergen, Norway.

The laboratory is accredited by ISO-standard 15189:2012. All analyses were on Roche e602/e801 (TSH and T4) or c702 (albumin and CRP). For serum TSH, we employed the laboratory-defined cutoffs for patients of age 20 years or older, defining TSH within 0.40 to 4.50 mIU/L as within the reference limit. Low TSH was defined as 0.40 or lower, whereas levels greater than 4.50 mIU/L were defined as high levels. For serum TSH ≥ 10.5 mIU/L (Roche Cat# 11731459, RRID:AB_2756377), the level was set to 10.5 to avoid outlier effects of the regression model to minimize unintended influence on the calculated mean values. Similarly, for serum T4 ≥ 30 pmol/L, T4 was set to 30 pmol/L (Roche Cat# 07976887190, RRID:AB_2861411).

C-reactive protein was included as a marker of inflammatory diseases. Some studies have demonstrated CRP to be elevated in thyroid diseases [29]. Serum CRP was categorized into 2 groups: ≤30 mg/L vs elevated levels >30 mg/L. The level was set at 30 mg/L to exclude patients with low-grade inflammation explained by aspects from substance use such as injecting substance use. Serum albumin was included as a marker of liver damage or disease as albumin is quantitatively the most important thyroid hormone-binding proteins in humans. High serum albumin may also be associated with hypothyroidism [30]. Serum albumin was categorized into 2 groups: <35 vs ≥35 g/L.

Serum TSH was used as the primary outcome measurement for thyroid disease in this study. Data on T4 was obtained as additional clinical information on thyroid function.

The values of TSH, T4, CRP, and albumin were obtained from the electronic laboratory database.

Four patients had a serum TSH ≥10.5. No patients had a serum T4 ≥ 30.0. A total of 13 patients had serum a CRP >30 mg/L and <5 patients had a serum albumin <30 g/L.

### Definition of study variables: clinical and sociodemographic factors

Analyses of serum TSH, T4, CRP, and albumin were performed in closest proximity in time to the respectively health assessments for each patient. The first serum TSH measurement was defined as the first assessment, whereas the subsequent measurement was defined as the second assessment. Time was defined as years from the first assessment.

Educational attainment was classed into the following groups: primary school (9 years), high school (12 years), and higher education (college or university). Substance use was defined categorically as using any of the following substances during the 12 months leading up to the annual health assessment: alcohol, cannabis, benzodiazepines, stimulants (amphetamines and cocaine) and non-OAT opioids (eg, heroine). Information regarding housing was collected within a range of 30 days from the date of health assessment. Housing conditions for the past 30 days leading up to the annual health assessment were defined as stable (living in owned or rented home or at an institution) or unstable (being homeless, living at shelter or with friends and family). We defined OAT dose ratio as ratio 1 = buprenorphine 18 mg or methadone 90 mg. The OAT dose ratio was set due to prescribed daily dose of medication divided by the mean of the World Health Organization's recommended dose interval (90 mg for methadone and 18 mg for buprenorphine) [1].

### Statistical analyses

**Figure 2:**
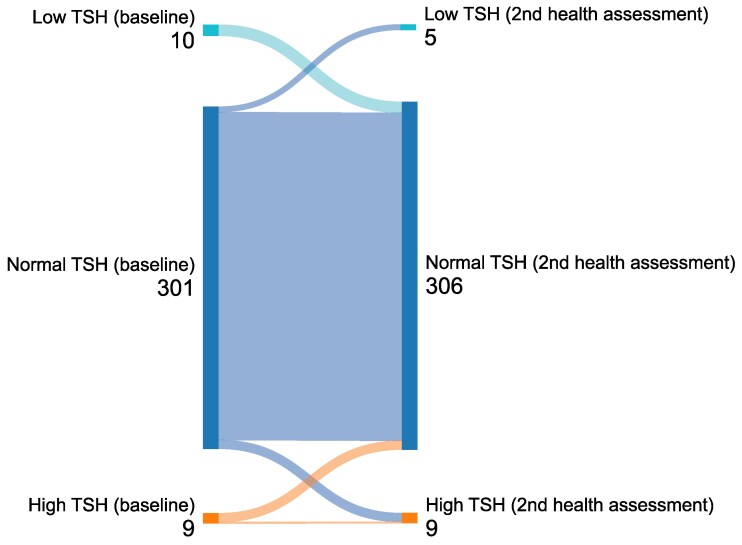
Sankey plot exploring the movement flows of serum TSH at baseline and the second serum measurement. Serum TSH was categorized in 3 levels: low (≤0.40 mIU/L), normal (>0.4-4.5 mIU/L), and high (>4.5 mIU/L).

Stata/SE 17.0 (Stata Corporation, College Station, TX) was used to generate descriptive statistics and a linear mixed model. SPSS version 29.0 (IBM, Armonk, NY) was used for expectation maximization imputation. Sankeymatic (sankeymatic.com) was used to generate the Sankey diagram (Fig. 2). Microsoft Excel was used to display descriptive data of the study sample. Descriptive data were presented with total numbers and percentages, including only valid, nonimputed values. The Sankey diagram was generated to visualize low-normal-high TSH over time from the first assessment. The threshold of statistical significance was set to *P* < .05 for all analyses. Time was defined as years from first assessment.

A total of 10.7% of values were missing across the predictor variables, including OAT medication and dose, substance use patterns in the past year (use of opioids other than OAT, stimulants, benzodiazepines, cannabis), housing status, CRP, and albumin. These missing data were considered missing at random, and expectation maximization imputation was performed to replace them with estimated values.

The linear mixed model analyses were performed to estimate associations between serum TSH or T4 (outcome variables) and the predictor variables, including gender, age, substance use, OAT medication, housing condition, educational level, CRP, albumin, and OAT dose ratio at first assessment, and to what extent they affected the outcome variables between the health assessments. Predictor variables were kept constant to the value held at first assessment. To assess the robustness of the findings, 2 sensitivity analyses were conducted: 1 excluding patients with TSH levels ≥10.5 in models where TSH was the outcome, and another excluding those with albumin levels ≤35 in models where T4 was the outcome, at any assessment point, to reduce the influence of potential outliers [29]. All models were random intercept fixed slope with the estimator set to restricted maximum likelihood. All available serum TSH or T4 measurements across time points were included in the analyses.

### Ethical approval

The study was approved by the Regional Ethical Committee for Health Research, Norway (REK no: 155386 and REK Vest 2017/51). Each patient provided written informed consent before enrolling in the study.

## Results

### Patient's characteristics at first assessment

Mean age was 45 years (SD 10) and 235 (73%) were men (Table 1). The most frequently OAT medication used was buprenorphine-based medication (51%), followed by methadone (47%) and morphine (1%). Eight percent lived under unstable housing conditions. Five percent reported primary school as their highest educational attainment.

**Table 1:** Descriptive characteristics of the study population at baseline (n = 320)

Age (years), n (%)	
18-25	8 (3)
25-40	108 (34)
40-60	184 (58)
≥60	20 (6)
Mean (SD)	45 (10)
Sex, n (%)	
Male	235 (73)
Female	85 (27)
Opiate agonist therapy medication, n (%)	
Buprenorphine	164 (51)
Methadone	151 (47)
Others	5 (1)
Mean dose in mg (SD)	
Buprenorphine	15 (6)
Methadone	98 (24)
Number of years in opioid agonist therapy, mean (SD)	8 (5)
Educational attainment, n (%)	
Not completed primary school	16 (5)
Primary school (9 years)	153 (48)
High school (12 years)	125 (39)
College or university (1 or more years)	24 (8)
Injected substances the past 6 months, n (%)	145 (48)
Unstable housing status the past 30 days*^a^*, n (%)	25 (8)
Substance use during the past year*^b^*, n (%) (n = 316)	
Cannabis	242 (76)
Benzodiazepines	228 (72)
Alcohol	225 (71)
Stimulants (amphetamine or cocaine)	175 (55)
Opioids (other than OAT opioids)	123 (39)

Abbreviations: NA, not available; OAT, opioid agonist therapy.

^*a*^“Unstable housing status” was defined as living in a homeless shelter or with family or friends at any time during the 30 days leading up to the health assessment. “Stable housing status” was defined as having owned or rented housing situation or being incarcerated during the 30 days leading up to the health assessment.

^*b*^The number of patients who had used substances at least once during the year leading up to the first assessment visit.

During the 12 months leading up to the first assessment, 307 (96%) patients reported use of cannabis, 294 (92%) patients had used benzodiazepines, 310 patients (97%) had used alcohol, 192 patients (60%) had used opioids other than OAT medication, and 179 patients (56%) had used stimulants (amphetamine or cocaine).

### Thyroid function tests at first assessment and over time

At first assessment, the median (interquartile range) serum TSH and T4 were 1.5 mIU/L (1.2) and 16.9 pmol/L (3.7), respectively. A total of 301 patients had a normal serum TSH, 10 patients had low serum TSH, and 9 patients had high serum TSH at the first assessment (Fig. 2). At the second assessment, the median (interquartile range) serum TSH and T4 were 1.9 mIU/L (1.1) and 16.2 pmol/L (3.2), respectively. Of these, 306 patients had a normal serum TSH, 5 patients had low serum TSH, and 9 patients had high serum TSH (Fig. 2). The proportion of changes between low/normal/high categories between the assessments was low. Excluding patients with serum TSH and T4 level above or under the reference range did not alter the results [29]. We also performed T4 analyses excluding patients with albumin levels <35 g/L; this exclusion likewise did not affect the results [29].

### Sociodemographic and clinical factors at first assessment and over time

No association between sociodemographic and clinical factors and serum TSH was found at first assessment and over time (Table 2). Further, adjusted for the predictors, the levels of TSH were stable over time (0.3; CI, −0.4 to 1.0). No differences in serum TSH between buprenorphine and methadone at the first assessment (0.0; CI, −0.3 to 0.2) and over time (0.1; CI, −0.2 to 0.1) were found. Patients with serum albumin lower than 30 g/L tended to have a higher TSH level at first assessment (0.2; CI, −0.9 to 1.3), with a tendency toward decreased levels over time (−0.2; CI, −0.7 to 0.4). Moreover, no association between sociodemographic and clinical factors and T4 was found at first assessment and over time [31].

**Table 2:** Linear mixed model of the impact of sociodemographic and clinical factors on the serum TSH (mIU/L) at baseline and over time (per year)

	(n = 320, number of observations: 640)	
	Effect estimate (baseline)	Time trend (per year) (over time)
	Coefficient (95% CI)	Coefficient (95% CI)
Time (per year)	—	0.3 (−0.4 to 1.0)
Sex		
Female	0.0 (−0.2 to 0.3)	0.0 (−0.2 to 0.1)
Age groups, years		
18-<25	0.0 (ref.)	0.0 (ref.)
25-<40	−0.2 (−1.0 to 0.6)	0.0 (−0.5 to 0.6)
40-<60	0.2 (−0.6 to 1.0)	−0.1 (−0.7 to 0.5)
≥60	−0.3 (−1.2 to 0.7)	0.1 (−0.7 to 0.6)
OAT medication		
Methadone	0.0 (ref.)	0.0 (ref.)
Buprenorphine	0.0 (−0.3 to 0.2)	0.1 (−0.2 to 0.1)
Morphine	0.5 (−0.8 to 1.9)	0.7 (−1.8 to 0.3)
OAT dose ratio*^a^*	0.0 (−0.1 to 0.1)	0.0 (−0.1 to 0.1)
Educational attainment		
Not completed primary school	0.0 (ref.)	0.0 (ref.)
Primary school	0.5 (−0.1 to 1.0)	0.2 (−0.5 to 0.2)
High school	0.6 (0.0-1.2)	0.1 (−0.5 to 0.2)
Higher education	0.1 (−0.6 to 0.8)	0.0 (−0.5 to 0.4)
Unstable housing status	0.1 (−0.4 to 0.6)	0.0 (−0.3 to 0.3)
CRP ≥ 30	0.0 (−0.7 to 0.6)	0.0 (−0.3 to 0.3)
Albumin ≤ 35	0.2 (−0.9 to 1.3)	−0.2 (−0.7 to 0.4)
Substance use in the past year		
Alcohol	0.1 (−0.2 to 0.4)	0.1 (−0.1 to 0.2)
Cannabis	−0.3 (−0.6 to 0.0)	0.0 (−0.2 to 0.2)
Benzodiazepines	0.1 (−0.2 to 0.5)	−0.1 (−0.3 to 0.1)
Stimulants (amphetamines and cocaine)	−0.1 (−0.4 to 0.2)	0.1 (−0.1 to 0.3)
Opioids (other than OAT medication)	0.0 (−0.3 to 0.3)	0.0 (−0.2 to 0.1)

Abbreviations: CRP, C-reactive protein; OAT, opioid agonist therapy.

^*a*^OAT dose ratio: 1.0 = 18 mg buprenorphine, 90 mg methadone, 350 mg morphine.

## Discussion

This study documented that the mean TSH values was within normal range among patients receiving OAT.

No associations between serum TSH and T4 and substance use patterns were detected among patients receiving OAT. This supports existing literature in this field. Few studies have assessed the impact of illicit substance use patterns on thyroid function; however, a dysregulated hypothalamic-pituitary-thyroid axis is found among patients with alcohol use disorders [32, 33]. One study found that thyroid function was dysregulated in men using methamphetamine [34]. Studies evaluating the impact of cannabis on thyroid function showed equivocal results with low evidence [35]. No recent studies have examined a possible association between use of benzodiazepines and thyroid function.

Furthermore, our results documented TSH values within the recommended range with substantially small changes over time. This points to the fact that screening for thyroid function with TSH is not indicated among patients receiving OAT. However, a meta-analysis from 2020 [36] recommended periodic evaluation of the gonadal axis in males and adrenal axis for patients on long-term use of opioids. Several mechanisms may explain why TSH values slightly outside the reference values normalize over time. Individuals with undiagnosed or fluctuating autoimmune thyroid disease may vary around the upper reference limit. Normal biological variation, particularly the relatively wide intraindividual variability of TSH compared with the more tightly regulated fT4, can produce substantial shifts, especially when samples are drawn at different times of the day [37]. Additionally, transient elevations in TSH may occur during recovery from acute illness. Together, these factors provide plausible explanations for spontaneous normalization as seen in some patients in our study. However, it should be a low threshold for testing based on symptoms because thyroid disorders are highly prevalent [38] and many patients attending OAT treatment do not seek health care.

Serum TSH was used as the primary outcome measurement for thyroid disease in this study. TSH is the primary marker of thyroid function because of the sensitivity to slightly fluctuations in serum T3 and T4 [16]. The exception is when the thyroid function is affected by insufficiency in production of thyrotropin-releasing hormone or TSH in hypothalamus (thyrotropin-releasing hormone) and hypophysis (TSH). One cause of insufficiency in TSH production occurs in hypopituitarism. However, it has been suggested that the thyroid axis is 1 of the last axes to be affected [39], and our results could fit with that.

One significant strength of this prospective study is the relatively large study population. The patient group enrolled in the OAT program accommodates many patients defined as “hard-to-reach.” This design made it easier to tailor the exposure data that could be relevant. All patients receiving medication for thyroid diseases or having a thyroid disease registered were excluded. We also had patient information on other endocrine diseases, but only information registered in the specialized health care. One main limitation of the study was related to the timing of health assessments and blood samplings. The health assessments were done almost annually, but the time span between the health assessment and blood sample could vary to some extent. Another limitation is that patient information on social aspects such as education level, housing, and illicit drug use was self-reported, which could impact the results.

## Conclusion

This study documented no association between thyroid function and substance use, clinical, or sociodemographic factors among patients receiving OAT. Few patients had values indicating thyroid disturbances. The results indicate that screening for thyroid disturbances beyond symptomatic assessment in people undergoing OAT is not needed. These findings underscore a compelling opportunity to refine clinical practice. Investigation on the interplay between thyroid function and opioid agonist therapy has been important to ensure evidence-based and patient-centered care.

## Data Availability

Original data generated and analyzed during this study are included in this published article or in the data repositories listed in the References.

## Abbreviations

CRP: C-reactive protein
OAT: opioid agonist therapy
